# Conversion for Severe Malnutrition After Single Anastomosis Duodeno-Ileostomy with Sleeve Gastrectomy (SADI-S) to Duodenojejunal Bypass (DJB): a Case Series and Literature Review

**DOI:** 10.1007/s11695-025-08357-8

**Published:** 2025-11-10

**Authors:** Sol Lee, Ina Chen, Mélissa V. Wills, Matthew Kroh, Ali Aminian, Salvador Navarrete

**Affiliations:** 1 Seoul Medical Center, Seoul, Republic of Korea; 2 Cleveland Clinic Lerner College of Medicine, Cleveland, United States

## Abstract

Single Anastomosis Duodeno-ileostomy with Sleeve Gastrectomy (SADI-S) is an emerging bariatric procedure that can sometimes lead to severe malnutrition. This report describes two cases of conversion surgery for post-SADI-S malnutrition and reviews management strategies. Both patients, who had undergone sleeve gastrectomy followed by SADI-S for weight recurrence, developed severe protein-energy malnutrition with hypoalbuminemia (1.6–1.8 g/dL) requiring parenteral nutrition, which normalized following conversion surgery. They underwent laparoscopic conversion to Roux-en-Y duodenojejunal bypass with common channel lengthening (Case 1: 200→550 cm, Case 2: 250→750 cm). Postoperatively, both recovered without needing parenteral support. Our technique provides significant clinical advantages through reduced operative complexity and avoidance of challenging duodenal stump management while achieving excellent nutritional outcomes. While this demonstrates the potential of surgical intervention in managing severe post-SADI-S malnutrition, the current absence of standardized protocols for post-SADI-S conversional surgery underscores the clear necessity for evidence-based treatment algorithms through multicenter research.

## Introduction

Single anastomosis duodeno-ileal bypass with sleeve (SADI-S) is a recently recognized metabolic and bariatric surgery (MBS) technique gaining traction in the United States [1]. Developed by Sanchez-Pernaute and Torres et al. in 2007, SADI-S is a modified version of the biliopancreatic diversion with duodenal switch (BPD/DS). In this technique, a sleeve gastrectomy (SG) is performed, with different bougie sizes depending on surgeons’ preference. The length of the common channel varies as well, but is accepted in the bariatric surgery community to go anywhere between 250 and 350 cm [1]. The 2020 International Federation for the Surgery of Obesity and Metabolic Disorders (IFSO) Position Statement recognizes SADI-S as a secure and well-established surgical intervention, demonstrating significant weight loss and metabolic benefits [2]. 

Despite these advantages, SADI-S can lead to nutritional deficiencies like other bariatric procedures. While technical modifications have been proposed to address these issues, standardized guidelines for determining when patients require surgical intervention remain absent [3, 4]. In severe cases, malnutrition may necessitate conversion surgery.

Understanding the pathophysiological basis of these deficiencies proves essential for clinical management. SADI-S induces malnutrition through several anatomical and physiological mechanisms: creating a significantly shortened common absorptive limb (250–350 cm) that forces all nutrients through this limited segment, bypassing the jejunum which contains the highest concentration of nutrient transporters, and altering bile acid circulation patterns that further impair absorption [3, 5]. 

This case series examines the outcomes and feasibility of SADI-S conversion surgery in patients with severe post-bariatric malnutrition. We describe a novel surgical approach that preserves duodeno-ileal anastomosis and creates a Roux-en-Y configuration with two small bowel anastomoses to lengthen the common channel. This intervention may provide insights into managing severe nutritional deficiencies following SADI-S. Our findings may contribute to developing evidence-based guidelines for addressing malnutrition and determining appropriate surgical approaches in SADI-S patients.

## Case Presentation 1

A 47-year-old female patient with a history of chronic obstructive pulmonary disease (COPD), hypothyroidism, gastroesophageal reflux disease (GERD), and cholecystectomy underwent bariatric surgery. Initially, she received a laparoscopic SG in 2018. Her body mass index (BMI) decreased from 60.8 kg/m^2^ to 33.19 kg/m^2^. Due to weight recurrence (BMI: 42.58 kg/m^2^), she underwent a revisional single anastomosis duodeno-ileal bypass with sleeve gastrectomy (SADI-S) in 2021 with a common channel of 200 cm. Post-SADI-S complications arose, attributed to a short common channel and inadequate follow-up.

In early 2023, the patient presented with a BMI of 26.9 kg/m^2^, recurrent admissions for bilateral lower extremity edema, weakness, and diarrhea. Clinical evaluation revealed chronic anemia (Hgb 6.4 g/dL), hypoalbuminemia (Alb 1.6 g/dL), multiple micronutrient deficiencies, and steatohepatitis with anasarca, confirmed by CT imaging. These conditions were attributed to malabsorption secondary to duodeno-ileal bypass. Treatment was initiated with total parenteral nutrition (TPN) and liquid vitamin supplementation. Despite these interventions, the patient’s BMI further declined to 25.24 kg/m^2^ upon admission, underscoring the severity of her nutritional compromise (Table I).

To address the patient’s severe malnutrition, a diagnostic laparoscopy was performed with the plan for enteral access to the proximal jejunum. The surgery revealed the existing SADI-S configuration: a single duodeno-ileal anastomosis bypass located 500 cm from the ligament of Treitz, with a common channel measuring 200 cm. As part of the procedure, a jejunostomy tube was placed 30 cm distal to the ligament of Treitz. Over the next three months, the patient received enteral nutritional supplementation via this jejunostomy tube. This intervention significantly improved the patient’s nutritional status, making it possible to consider surgical conversion from SADI-S to a proximal Roux-en-Y configuration. The albumin level preoperatively was 4.1 g/dL.

The patient had a 500 cm afferent limb and 200 cm common channel. The goal of the surgery was to incorporate 450 cm of the existing afferent limb into the common channel. The surgical steps involved taking down the feeding jejunostomy, measuring the entire bowel, dividing the ileum 100 cm proximal to the ileocecal valve, dividing the ileum a few centimeters proximal to the existing duodeno-ileostomy anastomosis, elongating the common channel by anastomosing the divided ileum proximal to the duodeno-ileostomy with the distal divided ileum (which was connected to the cecum), creating a Roux limb configuration by anastomosing the divided ileum (the segment 100 cm proximal to the ileocecal valve) to the jejunum 50 cm proximal from the ligament of Treitz, and closing the mesenteric defect. The final configuration, after creating two new side-to-side small bowel anastomoses, included a 100 cm Roux limb, 50 cm biliopancreatic (BP) limb, and 550 cm common channel, with bypassing of the duodenum and proximal jejunum (duodenojejunal bypass [DJB]) along with the existing SG (Figures I & II**).**

**Fig. 1:**
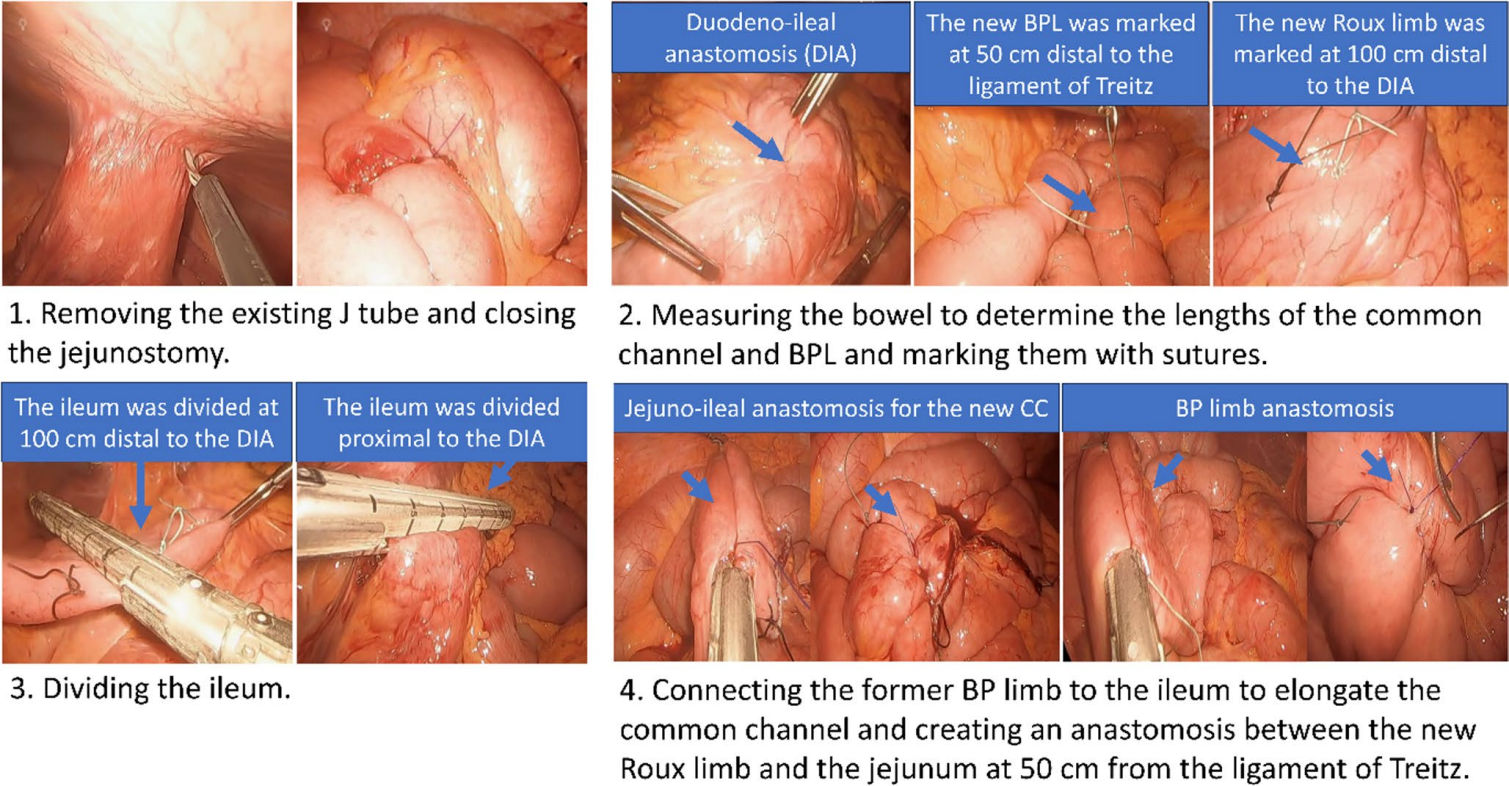
Intraoperative images of conversion of SADI-S to Roux-en-Y DJB while maintaining the existing duodeno-ileostomy

**Fig. II:**
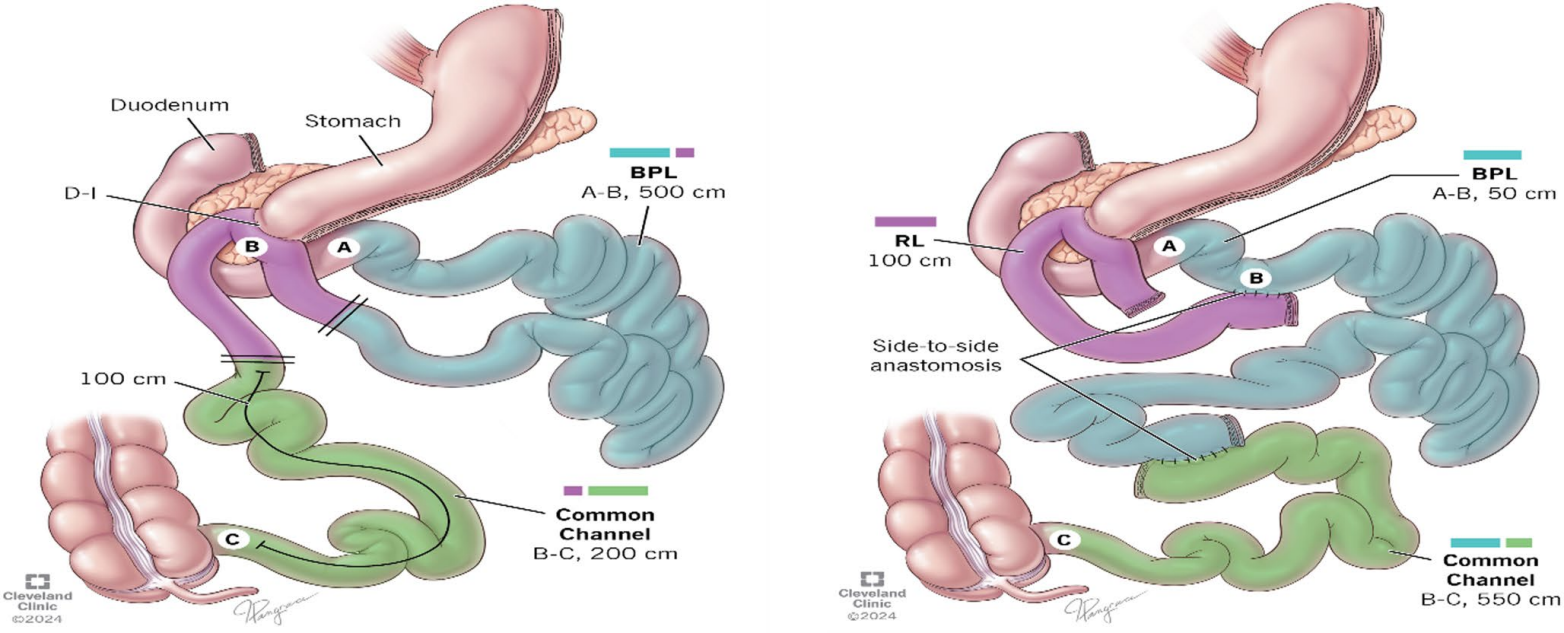
Conversion of SADI-S to Roux-en-Y DJB with 2 side-to-side small bowel anastomoses incorporating the maximum amount of Bilio Pancreatic limb to the common channel while maintaining the existing duodeno-ileostomy. Where A-B represents the original and final BP limb. Roux limb represented by purple color. B-C final common channel

The patient tolerated the surgery well, with minimal blood loss and an uneventful hospital stay. She was discharged on the second postoperative day. At the three-month follow-up, the patient maintained adequate nutrition through oral intake alone, and her blood chemistry values had normalized including Albumin and liposoluble vitamins.

### Case Presentation 2

A 48-year-old female with a history of hypertension, angina pectoris, obstructive sleep apnea (OSA), and metabolic dysfunction-associated steatohepatitis (MASH) underwent laparoscopic SG in 2017, with an initial BMI of 63.2 kg/m^2^. Her BMI decreased to 49.8 kg/m^2^. Following weight recurrence to a BMI of 57.9 kg/m^2^, she had a laparoscopic conversion of SG to SADI-S in 2019 with a common channel of 250 cm. Three months post-surgery, she began experiencing recurring episodes of leg swelling, leading to multiple ER visits. Eventually, she was admitted directly to the hospital due to worsening symptomatic malnutrition following her SADI-S procedure.

She presented with chronic iron deficiency anemia (Hgb 6.2 g/dL), hypoalbuminemia (albumin 1.8 g/dL), multiple electrolyte deficiencies, neuropathy, edema, and chronic diarrhea. Despite consuming regular food, she had required home parenteral nutrition starting 6 months after the malabsorptive surgery. Her BMI had dropped to 35.2 kg/m^2^, with a total weight loss of 89.4 kg since the SADI-S procedure (Table 1).

**Table 1:** Patient characteristics and conversion outcomes

Patient	Age	Initial BMI	PreOp BMI	Albumin (g/dL)	CC Length Change	Cause for conversion	Albumin level 6 month after.
1	47	60.8	25.24	1.6	200→550 cm	PCM with multiple nutritional deficiencies, steatohepatitis with anasarca	3.8 g/dL
2	48	63.2	35.2	1.8	250→750 cm	PCM with multiple nutritional deficiencies, chronic diarrhea	4.1 g/dL

*PCM*, protein-calorie malnutrition

In 2021, the patient underwent laparoscopic conversional surgery from SADI-S to Roux-en-Y DJB. Intraoperative findings revealed a loop duodeno-ileal anastomosis bypass 750 cm from the ligament of Treitz, with a 250 cm common channel. The procedure aimed to revise the loop DS to a Roux-en-Y DJB.

The surgical steps involved dividing the ileum 175 cm proximal to the ileocecal valve and dividing the ileum a few centimeters proximal to the existing duodeno-ileostomy anastomosis. The continuity of the small intestine was established by anastomosing the divided ileum proximal to the duodeno-ileostomy with the distal divided ileum (connected to the cecum). A new Roux limb was created by anastomosing the segment of small intestine that was attached to the stomach to the proximal jejunum 175 cm from the ligament of Treitz. Finally, the mesenteric defect was closed. The final configuration included a 75 cm Roux limb, 175 cm BP limb, and 750 cm common channel.

The patient tolerated the procedure well, with minimal blood loss (75 mL) and an uneventful hospital stay. She was discharged on the first postoperative day. At the two-month follow-up, the patient maintained adequate nutrition through regular oral intake alone, and her blood chemistry values had normalized.

## Discussion

SADI-S was developed to mitigate nutritional deficiencies and adverse effects associated with traditional duodenal switch operations [6–8]. It offers advantages over other malabsorptive bariatric surgeries, including reduced risk of fat-soluble vitamin and micronutrient malabsorption, shorter operative time, and lower incidence of internal herniation [1, 2, 6, 9–11]. However, malabsorptive complications may still occur in some cases [3]. 

The common channel length (CCL) is critical in determining the risk of nutritional deficiencies post-SADI-S. Currently, the optimal CCL to minimize malnutrition and optimize at the same time the best weight loss and metabolic response remains undetermined [7]. Initial experiences with a 200-cm CCL revealed high nutritional issues, prompting surgeons to extend the absorptive channel to 250–300 cm [12]. As we presented in this case series, two patients with common channels below 300 cm, presented with severe nutritional deficiencies. A 300-cm CCL has consistently demonstrated the most favorable outcomes with an acceptable malnutrition rate [13]. On the other hand, is important to perform a complete count of the intestinal length on each case, in the second case the bypassed segment of BP limb represented 75% of the entire intestinal length, leaving the patient with a 25% of absorption capacity.

Despite extended CCL, some patients may experience persistent hypoalbuminemia and other deficiencies, potentially due to inappropriate patient selection, limb measurement errors, or suboptimal compliance with dietary and supplementation recommendations [14, 15]. Recent studies have reported varying rates of vitamin and mineral deficiencies among SADI-S patients, even with supplementation [15, 16]. 

Our case series illustrates the challenges of malabsorptive bariatric procedures through two middle-aged female patients with similar surgical histories: initial SG, followed by malabsorptive procedures due to weight recurrence. The severity of postoperative malnutrition varied between patients, primarily attributed to differences in bypassed bowel length and follow-up compliance. While the malabsorptive component of the common channel was the main contributor to nutritional status in both cases, Patient 1’s poor adherence to nutritional protocols resulted in severe complications, including hepatic decompensation, massive ascites, and hypoalbuminemia. In contrast, Patient 2’s superior compliance with postoperative care led to milder complications.

Managing malnourished patients after SADI-S is an extremely time sensitive issue to be addressed before developing multiple organ dysfunctions and irreversible damages. Current guidelines for malabsorptive bariatric procedures recommend regularly monitoring patients at 3, 6, and 12 months post-surgery, followed by yearly check-ups [17]. If deficiencies persist despite supplementation and intermittent intravenous treatments, revisional or conversional surgery should be considered before severe or irreversible consequences develop [3]. 

Severe malnutrition following SADI-S can prompt consideration of various surgical management options, including conversion to single anastomosis duodeno-jejunostomy with sleeve gastrectomy (SADJ-S), SG with reversal of the malabsorptive component, or Roux-en-Y gastric bypass (RYGB). All these procedures would require revising of existing duodeno-ileostomy anastomosis (Table 2). Handling the duodenal cuff in these situations can be challenging. The selection of a specific procedure hinges on individual patient needs and anatomical factors [3, 6]. In our case series, both patients underwent conversion to Roux-en-Y DJB, albeit with differing BP limb lengths. Patient 1 (BMI 25) received a shorter BP limb, while Patient 2 (BMI 32) had a longer BP limb. In converting SADI-S to Roux-en-Y DJB, the existing duodeno-ileostomy is kept intact. Instead, two side-to-side small bowel anastomoses are created to address malnutrition incorporating the maximal amount of biliopancreatic limb to the common channel, leaving a 50 cm BP limb. From technical standpoint, this technique has several advantages: the preservation of the duodeno-ileal anastomosis protects the stomach from marginal ulcer a common complication after bypass modification, avoids working on a short duodenal cuff, also avoids dissection at the duodenal stump in case of complete reversal, optimizes the intestinal length of the common channel without the need for intestinal resection, decrease the complexity of the revision by doing small bowel anastomosis with known low morbidity and minimizes the bile reflux by dividing the ileal loop right before the duodeno-ileal anastomosis.

**Table 2:** Types of conversion surgery after SADI-S

Conversion Type	Surgical Technique	Advantages	Considerations
RYDJB (Our technique)	Division of afferent limb before duodenoileal anastomosis, creating Roux-en-Y configuration Incorporates majority of the BP limb to the common channel	Preserves original anastomosis, reduced operative complexity, avoids duodenal stump management	Require careful bowel measurement
Reversal to SG (3)	Duodeno-duodenal, and ileoileal anastomosis reconstruction	Most physiological restoration	Complex duodenal stump dissection, requires taking down original anastomosis
RYGB (18)	Gastrojejunal anastomosis with Roux-en-Y configuration	Reduce bile reflux complications. Relative simplicity of reconstruction	Requires taking down the duodeno-ileal anastomosis
OAGB (19)	Single gastrojejunal anastomosis	Simpler than RYGB, maintains single anastomosis	Risk of bile reflux
SADJ-S (3)	Duodenojejunal anastomosis at remaining duodenum	Simple, maintains one anastomosis advantages, short operative time	If anastomosis site < 1 cm from pylorus, Roux-en-Y conversion preferred

On the other hand, after six months of follow-up, both patients had normalized their albumin and vitamin levels, demonstrating the efficacy of this technique in the medium term. 

The approach to revisional surgery following SADI-S lacks standardization, with technique selection rationales varying globally. A key challenge in assessing individual patient needs stems from variations in small bowel length [18]. Consequently, comprehensive small bowel measurement is crucial for appropriate procedure selection and determining the extent of proximalization [4, 14, 19, 20]. This individualized approach underscores the complexity of managing post-SADI-S complications and highlights the need for tailored surgical interventions.

Despite these challenges, several studies have reported the safety and feasibility of revisional operations following SADI-S [3, 4, 14]. Our patients’ successful outcomes underscore the potential of these operations as a viable option when conservative measures prove inadequate. Future research should focus on developing standardized guidelines for managing nutritional deficiencies and determining optimal timing and approach for surgical intervention in post-SADI-S patients.

## Conclusion

The cases presented demonstrate the efficacy of conversional laparoscopic surgery in managing severe post-SADI-S malnutrition. Converting to Roux-en-Y DJB with proximalization enabled nutritional recovery, while offering technical advantages over traditional reversal procedures. This review underscores the importance of individualized treatment plans, close monitoring, and timely intervention in managing complications following SADI-S. Future research should focus on developing standardized guidelines for conversional operations in SADI-S patients to optimize decision-making and long-term outcomes.

## Data Availability

No datasets were generated or analysed during the current study.
